# Video Q&A: Patients leading the direction of clinical research - an interview with Paul Wicks

**DOI:** 10.1186/s12916-014-0118-1

**Published:** 2014-07-18

**Authors:** Paul Wicks

**Affiliations:** 1 PatientsLikeMe, 155 2nd Street, Cambridge 02141, MA, USA

**Keywords:** Participant-led research, Personalized medicine, Patient reported outcomes

## Abstract

In this video Q&A, we talk to Paul Wicks about the emergence of participant-led research, and discuss how this field may be expected to develop in the near future, particularly with regard to personalized medicine.

## Introduction

Paul Wicks is a keen advocate on the power of personalized medicine in improving health outcomes. In his current role as Vice President of Innovation at PatientsLikeMe, he is responsible for the scientific and medical validity of the research platform of PatientsLikeMe. As an neuropsychologist, he leads a team of scientific researchers, who generate insights from the personal health data shared by patient members. Prior to joining PatientsLikeMe, Dr Wicks worked directly with patients from around the world to study cognition in rare forms of motor neuron disease (also known as amyotrophic lateral sclerosis (ALS)) and the psychological consequences of Parkinson’s disease.

In this interview (Additional file 1), we talk to Dr Wicks about the work produced by the PatientsLikeMe R&D team under his direction, which includes numerous peer-reviewed publications in major scientific journals. We also discuss the possible future directions of this evolving form of clinical research.

## Edited transcript

### 1. Tell us a bit about yourself and how you became involved in clinical research driven by online patient communities

I’m a neuropsychologist by training, and I started out my research career investigating cognitive aspects of motor neuron disease – or ALS, as it’s known in the US. At that time in the early 2000s, the Internet was growing, was becoming more widely used, and was no longer just for hobbyists and academics. In 2002, I took over the running of a website at Kings College Hospital, called “BUILD” – Building User Involvement in Motor Neuron Disease. That project had been set up by a nurse as a research project, to try to gather more patient information about how we could improve the services that we were offering at the hospital. They tried a variety of different ways of engaging with patients; there were real-world support groups, there was a newsletter, there was a phone line, and there was the online message board. Actually, it was the online message board that proved the most sustainable over time.

When the research funding came to an end, we were looking for volunteers to keep it running to some extent. There were two young-onset ALS patients, just in their 20s, talking to one another. I knew a little bit about the web at the time, so I thought I’d take over and volunteer. In my evenings, I would moderate this website and people would ask questions like: *“Why do we have to have placebo in randomized control trials?”* I was just a student, so I didn’t know. So I would go and ask the head of clinical trials, and she would tell me, and I would tell them. I ended up becoming this sort of translation layer between medical science and patient understanding. That’s something that I’ve continued with to this day.

### 2. PatientsLikeMe is one of the first examples of a patient network that has taken control of information exchange about research into their diseases. Can you explain how this works and how you got involved?

Back in 2005–2006, PatientsLikeMe was just being started by a family affected by ALS. They were trying to come up with ways to help their brother, Stephen Heywood, who was diagnosed with the disease when he was 29. The idea was that rather than just having to rely on the evidence base for symptom management (which at the time was very thin), what if people shared their experiences to say, “*Well this dosage of amitriptyline worked really well for excessive saliva, but it also caused constipation.”*? If people could share that sort of information systematically, then perhaps we could do research with it; perhaps we could do scientific analysis.

Some of the users of BUILD were the beta testers of PatientsLikeMe, and it was through that initiative that I came into contact with the Heywood family. I reached out to them and said what an interesting idea I thought this was, and they asked if I would moderate their forum. So I took what I’d been doing in the UK and translated it over to PatientsLikeMe.

ALS was the first condition discussed on PatientsLikeMe. We give people the opportunity to measure their condition by giving them patient-reported outcome measures and let them track their treatments against that. They can add information on prescription treatments, supplements, over-the-counter treatments, exercises, as well as the symptoms that they’re experiencing, either through their disease, or they can add other diseases, other symptoms. They can also add lab measures – things like their forced vital capacity or blood tests etc.

The real difference between a system like PatientsLikeMe and a clinical registry is that all the members of the system can see all of the data of everybody else. If you think of a personal health record that was online, it might be just between you and your doctor. Or if you think of something like LinkedIn, you have to give permission before two profiles can connect. It’s not like that on PatientsLikeMe. On our system, everybody can see everybody else’s data. So this is a very different approach that is more about openness and sharing, rather than privacy and protection.

What that allows is much more rapid research, much more rapid discovery. We’re asking questions in a scientifically rigorous way. We think about: what are the right questions to ask? What are the meaningful questions that patients can answer about their condition? What are the things which they don’t have insight into? For example, you don’t know your blood pressure right now, but you know how tired you are. So, we have to focus on what the right questions are that we can ask and what’s really relevant for the condition.

### 3. How, in your opinion, are patients starting to influence clinical research? Are there any examples?

There’s ‘patient-centered research’, which takes the existing research infrastructure and brings patients in. So it says: we’re going to do an observational study, we’re going to do a clinical trial, we’re going to set up a patient advisory board, etc. We’re going to ask selected members with this condition, or caregivers, to come and comment to give us advice on different things; for example: what are the most important research questions here, or do people really understand this outcome measure, or how can we make sure we communicate things clearly? I think that’s very important, and I think that’s long overdue.

**Figure 1:**
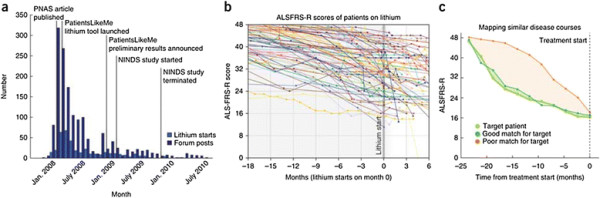
**Conceptual overview of the online study environment and matching algorithm. (a)** The number of patients choosing to experiment with lithium carbonate peaked in the months after publication of a small clinical trial in Italy. Preliminary negative results from this patient-led study were announced before the first randomized control trial had started recruitment. **(b)** Aggregate view of FRS scores for all 348 patients analyzed in this study. These data were publicly available online during the study. **(c)** Illustration of disease progression curves of control individuals who are good and poor matches for a particular patient. Both control individuals would be considered comparable by traditional matching criteria. The PatientsLikeMe matching algorithm minimizes the area between the disease progression curves for a target patient and a control. Adapted from [[6]] with permissions.

“Participant-led research” is slightly different. That’s saying: no, we’re not going to use the existing infrastructure of research, like hospitals and trained scientists, etc. We’re going to take some of those skills and transfer them over to the participants themselves. One example of that would be the lithium study that was conducted in about 2008–2009 by a group of people affected by ALS (Figure 1). They were responding to a clinical trial which had been published in the peer-reviewed literature (in PNAS [*Proceedings of the National Academy of Sciences*]), by an Italian group which claimed that lithium carbonate – a drug frequently used for the treatment of bipolar disorder – would halt or significantly delay the progress of this rapidly fatal, incurable disease. The title of the paper even said, “*Lithium delays amyotrophic lateral sclerosis.”* Members of our site translated the abstract out of the original Italian, using Google Translate. They were already trying to work out the best protocol for getting hold of this drug, before the medical research establishment had even read the published findings.

What ended up happening was, in the original Italian study they had got 16 patients treated with lithium carbonate, and they compared those with 28 controls who weren’t taking it. Within about six months, there were 160 patients who had got hold of lithium off-label – so that’s 10 times the sample size in the published literature. Led by a Brazilian patient, Humberto, and a caregiver in the States, called Karen, they started crowd-sourcing the data. One of the oddities about that disease is that the rating scale which is used in clinical trials can also be self-reported. Correlations between clinician interview and patient self-report is very high – it’s above 0.9 (on the ALSFRS-R [Amyotrophic Lateral Sclerosis Functional Rating Scale–revised] scale). What that means is, the patients have access to almost the same quality resolution of information, of measurement, that the doctors did.

So they started using Google Spreadsheets to exchange their experiences with lithium, to compare their functional rating scores, dosage and side effects. They started running statistical tests on their data. In particular, Karen had a PhD in geology, which is not a medical science, but it’s still a science. She was running *t*-tests to compare the outcomes in people who were self-reporting that they were taking lithium vs. a control sample who were not.

I think this raises a number of interesting questions, but one thing that I think can’t be overlooked is that the members, the patients, the participants were asking many of the right questions. They were analyzing the original study that had been published in PNAS and saying, “*I’m not sure about the confidence intervals on this graph,”* or, *“What was the dosage here?”* or *“Is this the right statistical test that they used? And was this single-blinded or double-blinded?”* They were picking the study apart just like a peer reviewer would. I think we often don’t give participants and patients enough credit for being able to do that.

In the end, what happened was that we upgraded our platform on PatientsLikeMe, to allow people to more systematically measure these things so we could break out not just the total score but also the component items; we could more rigorously capture longitudinal data over time; and we could capture data in a way that was probably a bit more robust than Google Spreadsheets. We had a dialogue open with the community the whole time about where we were with the analysis, and people gave us suggestions. When it came time to publish the paper, we published it in an open access journal so that all the members of the community could read it. Another thing that we did was we actually uploaded the entire de-identified dataset to the community, so that other people wanted to take that data and re-analyze it, they could.

### 4. Are there any risks to this type of research?

Historically, there have been signs of people perhaps rebelling against the system. In some cases, people with HIV in clinical trials would register at multiple clinics to try to make sure they were getting the treatment, not the placebo. Sometimes they would even meet up in person and swap pills; they would have “pill parties” so that people would get equal chances of placebo and treatment. The medical establishment thinks that the purpose of a trial is to learn something, but I think that these behaviors were an early indication that many patients think that the purpose of a trial is to get better. When these two things come into conflict, there’s some tension there.

What the Internet and some of the tools that technology has allowed is for the patients to actually become more on parity with the medical establishment. If they have access to some of the same tools — e.g. online courses, Wikipedia, open access publications – then I think we have to react to that. Otherwise, there are some risks that if we don’t react and we don’t build constructive ways in which we can harness this energy, it’ll actually backfire – it’ll actually be counterproductive – for both patients and the research field.

### 5. How will current ethical guidelines for research need to adapt to participant-led research?

I think, relative to our understanding of ethics as they exist in the developed world, in response to the Declaration of Helsinki, the Nuremberg trials and the Belmont report, participant-led research turns that on its head. Those reports and those ethical rules assume that there is such a thing as a researcher and such a thing as a participant, and that there’s never a situation in which one person can be both things. I think it assumes an imbalance of power where one person knows more than the other, has access to more social capital and somehow controls the other person. I think there’s a lot of stuff in there around protection of prisoners, of vulnerable people, that sort of thing. It doesn’t really take into account what can happen when the people themselves who have a condition want to self-experiment. I think we really need to think of how a co-production model of research is different here.

I don’t think there are any easy answers, but for instance in the lithium study, one of the main people who was proposing the patients do it was a patient living in Brazil, using Google Translate to communicate with English-speaking people around the world. Which IRB [institutional review board], which ethics committee is responsible for that? Lithium carbonate, at the time, was generic. It was made by dozens of manufacturers and it cost pennies. Is there a manufacturer that should take responsibility for that? Was the responsibility for each individual patient down to the doctor who wrote the prescription, because they were off-label prescriptions? Does some responsibility lie with PatientsLikeMe as a place that collected the data and published the research? I think that’s all unclear. But I think until the research establishment has more of a discussion about this, we’re not really sure where to go.

I think what you can’t do is close it all down; you can’t put the genie back in the bottle. Because if it weren’t PatientsLikeMe, it would be Google, or it would be Twitter, or it would be Facebook. You can run participant-led research over the telephone. The medium is not the issue here. The issue is this social activation that’s occurred and the really simple group formation that social media allows. Unfortunately, sometimes these things get tested when things go wrong. So we’ve been very carefully keeping an eye on other experiments that are going on in our platform and in the world more generally.

### 6. So, if patients start to take more control of the direction of research into their disease, how might this affect the patient-physician relationship?

In terms of the relationship with the clinician, I think it really varies with the condition to some extent. If you have quite a specialist condition like a neurological disease or a cancer, for example, frequently you’ll be dealing with a clinician who’s often involved in research. So I think they will take a keen interest in participant-led research, and my hope is that through clinician interfaces to these patient platforms – whether it’s PatientsLikeMe or somewhere else – the clinician will use that tool to keep an eye on their own patients.

The other very important space we have to think about is where clinicians aren’t involved in research. For instance, if you, as a relatively healthy person, are dealing with something like insomnia, diabetes, your weight, etc. – you probably are already self-experimenting. You cut out caffeine, you take up running – you do whatever it is that you have to do. So, there is some degree of self-experimentation already going on. I think the question is: at what threshold do we need to start informing people about what we’re doing, what we’re experimenting with?

Certainly, if you’re using a drug, that crosses a clear threshold. But what about dietary changes, what about supplements, what about some levels of exercise? I think there’s probably a point at which we need to either encourage patients to tell their clinicians or encourage clinicians to ask their patients: “*What else are you trying?”*

We certainly know in cancer and other neurological diseases that a fairly substantial minority of patients – around 15 to 20% – are taking off-label or complementary and alternative medicines and not telling their doctors. In the case where that’s a multivitamin, that’s one thing. But it’s a more serious issue if the patient is taking St. John’s wort or something that’s psychoactive or that could potentially have an interaction with a prescribed drug.

It’s somewhat dependent on the condition, but we also have to be mindful of the clinician’s attitudes to this. I think there are some things they could say that could potentially damage that patient–physician relationship. And this may not be for everybody; I would predict that some clinicians will naturally attract patients who are willing to self-experiment, and other clinicians will say, “*No, I need to be able to control the parameters, control the variables, because I am ultimately responsible for your health and, if you are going to self-experiment, then I’m not sure we’re going to be able to work together on this.”* That’s a different model. That’s saying that we’re tackling this condition together.

I think most doctors who are forward-looking and progressive would think of the problem that way. Certainly, people who work in terminal illnesses have been familiar with this for quite a while.

### 7. Are there any other challenges or limitations this form of research? For example, levels of bias?

**Figure 2:**
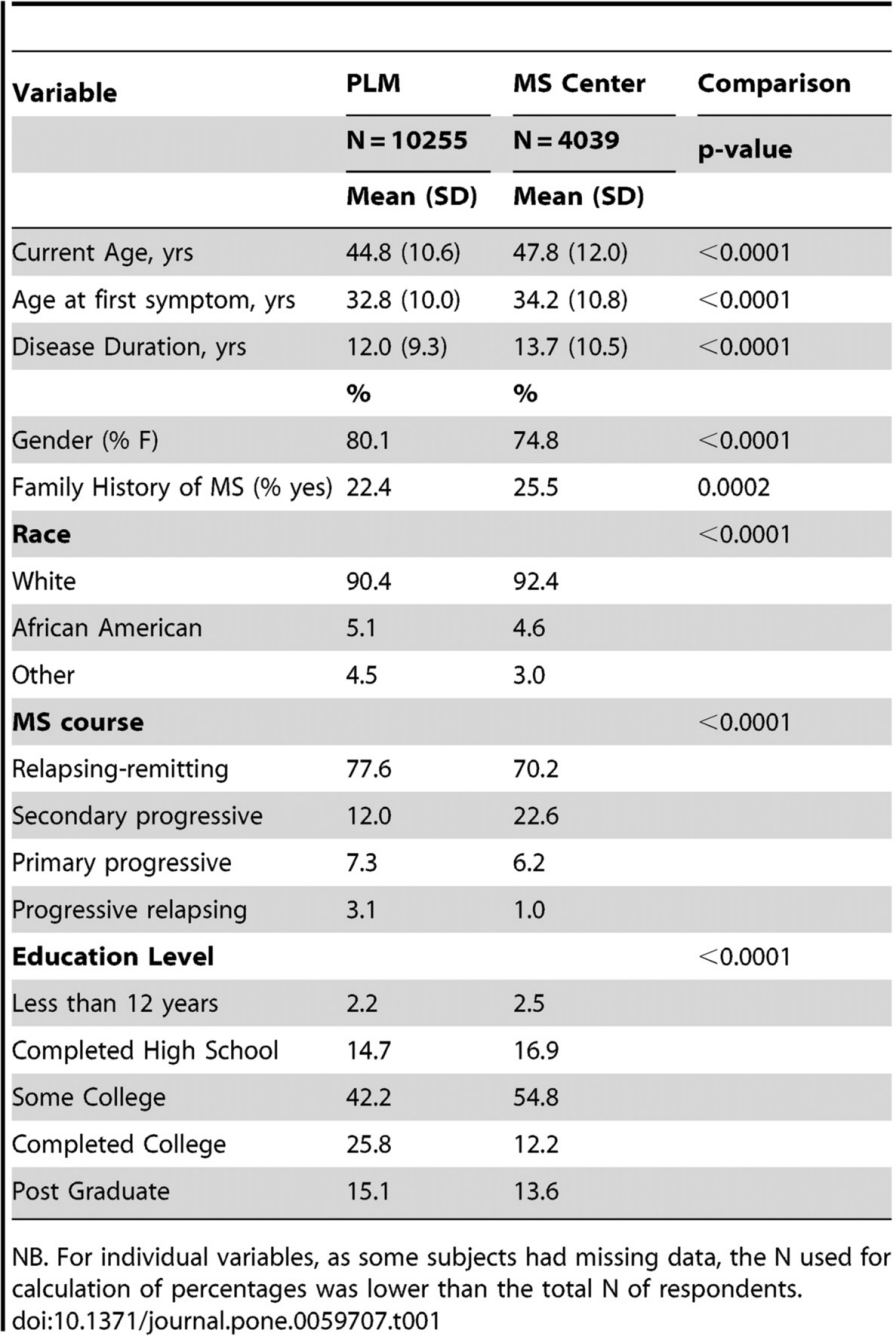
**Comparison of the individual and disease characteristics of the PatientsLikeMe.com members with those of patients followed at the Partners MS Center.** Adapted from [[16]] with permissions.

In terms of the challenges that we have to face, I think there are a few things that are common to all research projects. One is bias. Certainly, when we’ve compared the PatientsLikeMe sample, for instance in multiple sclerosis, with a tertiary research centre in Boston that sees thousands of patients, we found the group of people who self-report and join PatientsLikeMe tend to be younger, are more likely to be female, and tend to be slightly better educated. But the important point is – not to the extent that you might think (see Figure 2). They are a couple of years younger. They’re a few percentage points more likely to be female. This is not such a big bias that you can’t override it either by stratified sampling or by sample weighting. So these factors are addressable.

The other question is – compared with what? We’ve looked at data in organ transplants, for instance, where in the United States every organ transplant patient is catalogued, barcoded, and followed up in rigorous detail. Most diseases don’t have this level of detail recorded. So when someone asks if our population of patients with fibromyalgia is biased, well, we’ve got data points from 43,000 members in our system (last updated on June 2014). There are no other databases that large – so how would I know? If I’m biased relative to the consecutive case series of 120 patients in the literature – how do I know that they’re not biased? It’s very hard to tell. So that’s one of the issues that we have to solve.

The next issue is around a classic problem: on the Internet, no one knows you’re a dog. Well, on the Internet, how can anyone be sure that you’re a patient? You might have multiple sclerosis; you might think you have multiple sclerosis; you might not have multiple sclerosis but you’re trying to influence the community for some nefarious purpose or just because you’re messing around. Who knows? I think the degree to which we need to solve that depends on what problem we’re trying to solve.

If we’re going for a very rigorous high-end question like – should a regulator or a payer decide whether or not this drug is worth approving? – I think the data on which that is based have to be from people who’ve given some other level of verification that they are who they say they are. That could be as high as their doctor has signed a form, or it might not have to get quite that extreme. I think as people’s access to their medical records gets simpler, you could imagine them giving permission to other systems to use that data. In the same way that you have permissions around what the government can see about you, and what your bank can know about you or transfer to other banks under agreements like Direct Debit, for instance, you could imagine being able to determine the circumstances under which your personal information can be shared with other organizations– whether that’s universities or industry. That would allow us to upgrade the quality of the data, but still allow users and participants to retain a level of control.

The third issue is just familiarity. The people who make decisions about diseases, treatments, or health systems need to get more comfortable and more familiar with this sort of data. That’s why I think it’s very important to publish in the peer reviewed literature and to publish in open access journals where possible, so that there aren’t barriers to people reading these studies. It’s important to try out different data sources. Real-world data sources are messy data-wise – it’s certainly not as clean as a clinical trial, but it may be more generalizable.

I think that’s another place where there is both a limitation but a potential strength: data taken through online systems, or digital systems, may or may not be generalizable, depending on the condition. Those biases that I mentioned earlier don’t do very well if what you’re ultimately trying to study is, for example, schizophrenia in people who are homeless. You’re going to struggle to do that. But if your research question is around working-age women with multiple sclerosis, well, those demographics of younger and female actually work to your advantage. So I think it’s a question of actually picking out those populations where the bias and generalizability can be addressed, and looking at the status quo – where the databases and the samples are quite small.

### 8. Recently, Open Research Exchange (ORE) was created to facilitate collaboration between clinical researchers, academics, and patients. How does ORE expect to improve health outcome measurements?

A big part of building a learning healthcare system is about building the correct measurements. We can only look after our weight because we have weighing scales and there are labels in our clothes that tell us how big we are. For most of the thousands of medical conditions that exist, there are no such measures. You can’t line up a row of people from end to end and say “this is the person with the worst autism or OCD for instance, and this is the person with the least worst” in such a way that is anywhere near as scientifically and psychometrically as rigorous as we can with blood pressure or weight or height, or anything that’s more measurable.

This can be addressed to some extent. There are patient-reported outcome (PRO) measures, where through just questions and surveys, patients can tell us about their experience of a disease. That’s particularly important about things like pain, about symptoms, about quality of life, or about the impact of disease on the activities of daily living. Perhaps we can have an objective measure in something like rheumatoid arthritis from a blood test, but what we’re really interested in is: “*how well can you open a jar?”* or *“how well can you function in the real world?”* PROs really allow you to do that.

The issue, however, is that there aren’t many PROs. There’s the PROQOLID [Patient-Reported Outcome and Quality of Life Instruments Database ] that estimates there’s a little under 1,000 PROs, and if there’s about 7,000 diseases in ICD [International Classification of Diseases] and about 7,000 rare diseases that don’t even have names, then we need more measurements. The other issue is that many of these measurements were developed a long time ago, so they suffer from anachronisms; they say, “*how full of pep are you?”*; they ask, in an Asperger’s screening instrument, whether or not you can program a VCR [video cassette recorder]. In this world of TiVos and DVRs [digital video recorders], that’s no longer relevant.

What we need is a way of developing PROs faster. We could also do with reducing limitations on them. Many of the PROs are licensed, copyrighted instruments – you have to pay thousands of dollars for a license fee or several dollars per use, and that limits their use. It restricts their use in innovation and in app development, and restricts them only to expensive and well-funded clinical trials.

The point of the Open Research Exchange – funded by a grant from the Robert Wood Johnson Foundation – is to overlay a platform on top of PatientsLikeMe, which allows researchers to come and enter their prototype PRO measures, and for patients on our system to come and help to improve them. So, for instance, if there’s a question like, “*How frequently do you experience suicidal ideation?”* we can target a patient who we know has had a mood disorder in the past to come and provide feedback on that question. They might say something like, “*Well, gosh, I don’t know what ideation means. Do you mean have I had thoughts about killing myself? That’s a question that I can relate to*.” We can get more patients to look at that quickly, have the researcher iterate on that question, and then get another group of patients to come and provide further feedback. So we can very quickly improve upon individual questions.

You could also field it to a larger sample – to a few hundred people who are willing to be volunteers so that psychometric information can be gathered rapidly. So through item response theory we could ask, do we see enough segregation between the people saying “*not at all*” and “*a little bit*” or “*somewhat*”? Are these the right categories or should we use a 1 to 7 scale, or a 1 to 5 scale? There are psychometric statistical answers to that, but we frequently don’t get them in medicine because people don’t have access to a sample that can try out one version with five items and one version with seven items. However, because these people are online, they have the time to do that and they’re willing to do that.

In 2013, the mission was to develop three PRO measures rapidly and so far we’re on track to achieve that in a number of different areas. All those instruments will be licensed under a Creative Commons Sharealike licence, which means that they can be built upon and translated, and what we’re trying to build is an ecosystem around which PRO developers can come in and volunteer to help out other people. So, if you have a statistical skill and a little bit of spare time, or if you’re a good item developer, you could help your peers. We could create item banks and libraries of instruments so that if someone comes in, for example a parent who’s very well motivated and wants to develop a PRO for their child with a developmental disorder that has some characteristics of autism and Down’s syndrome, for instance, we could take those items that have tested well and build that into a new scale. Because it’s all under Creative Commons, we can reuse that work without having to worry so much about that copyright, and then go on to build new instruments.

So, it’s sort of an open library. Our software engineers talk about it as GitHub for PROs, which is a software development tool that allows a lot of open access work. *Next year [in 2014]*, what we’ll be looking at is trying to bring in more of those patients, more of those caregivers. When you let the software take off some of the heavy lifting, you’re able to concentrate on what you actually want to do. In this case, that would be: share your experience of what it’s like to live with a disease and give feedback to the researchers on what it’s actually like to be a patient.

### 9. How do you expect this field to develop in the next few years?

My hope is that people will be open to some of these ideas because, who knows, one of our participant-led research projects could be right. It could be successful. I think much of the existing scientific endeavor is set up around new, patentable pharmaceutical molecules. But in the case where it’s exercise or diet or something like that – maybe even a behavioral modification – there’s not a large well-funded machine behind it that can go and study that, which means that we can miss out. There are many conditions – diabetes is a perfect example – where those things can be just as effective, if not more so, without the side effects. But unless we build a system that captures that and allows it to filter back into the medical system, it’s going to remain just a sideshow on the outskirts. The next few years, I’m really expecting the governments of the world to wake up to this, for the health systems of the world to wake up to this, and to start engaging a bit more with the problem.

I think a lot of the participant-led research world has come from patients and participants, as you would expect. Frequently, people who are professionals in their working lives then get sick and have come from a very high-tech environment like architecture or engineering or town planning, and then they get ill and they’re amazed that these systems don’t exist and so they go and build them. That’s fantastic, that’s great, it means they come from exactly the right place. But at some point, these things need to scale up. They need to start interacting with the health system, and the health system is controlled by a variety of large players and big barriers to entry, and they are sometimes a little bit reticent to innovate.

I think the opportunity here is to say – well some of this work has been done. Some of the early barriers have been overcome by the pioneers, the patients out there in the field. I think now is the time for the people who run hospitals or run decision-making bodies, to say – well OK, what are some of the tendrils we can extend to see if we can link up here? Not to say – is it good enough yet? Pass or fail. But to say – well if these people did a, b and c, then we would be able to use their data, then we would be able to help involve them in the decisions more. Then we’d really be able to build their system into ours and make use of that.

Certainly, people with rare diseases have got a jump on this because the patients are so well motivated. But also, there are so few researchers involved in this that really they’re interested in all the help they can get. People like the Alkaptonuria Society (AKU), have been extremely involved in building clinical databases of patients all over the world, centralized out from the UK. They’re helping to run trials. They’ve even crowd-funded the recent replication of a trial; when their funding gap of $100,000 showed up, they didn’t just shut the trial down, they went out on IndieGoGo and started fundraising through their researchers, through their communities, through their hospitals, in the same way as some of Kickstarter commercial projects.

There are some interesting new ways that this can be harnessed and built into the existing research infrastructure. One of the most pioneering groups in this field is the Multiple Myeloma Research Foundation over in the States, who’ve actually worked very closely with the pharmaceutical industry, and even funded pharmaceutical companies to develop drugs. Now, that is exactly the opposite way round of how we normally think of a non-profit organization or a charity. But they’ve taken a very aggressive approach in wanting to look at treatments that have potential and to say – well even if there’s not an addressable market for this, we would like to make that investment from where we sit, to try and make use of the pharmaceutical industry’s existing infrastructure to do the science, to do the trials and to run those. I think we’ll see a little bit more of that; the existing infrastructure bringing in what’s being done out there, and sometimes even inversions of how we normally think of the paradigm. So I think those are two big trends we see.

The one trend I would love to see more of is where we see innovation in the developing world come through into the developed world. There’s a term “reverse innovation”, which I’m a bit ambivalent about. But the idea there is that you look at interventions that are cheap, that can be open sourced. There’s a great project called Fittle, run by an eye doctor in India who is 3D-printing toys with Braille. So you have something like a plastic fish, it’s shaped like a fish and it’s in four components and it’s got the Braille for F, I, S, H. He’s open sourcing it so that anyone can print it anywhere. This is a development that if a company came up with it, it would be very expensive, very complicated and would have to be ordered from a catalogue, etc. But this philosophy is saying – no, if you’ve got a 3D printer, it’s just intellectual property, you can just make it appear and it just costs pennies for the plastic mould. This sort of stuff is happening with prosthetics. It’s happening with assisted devices. It’s becoming easier and easier to do this. That’s a really important trend we have to look at.

Innovation is not just spending millions of dollars on big expensive technogadgets that go ‘ping’. Sometimes, it’s getting cheap little bits of plastic and wire, and roping them together with a bit of know-how, and then distributing and assimilating that knowledge in a way that’s low-tech, survivable, robust, and cheap, so that more and more people can have access to and use.

### 10. Where can I find out more?

See references [[1]–[21]].

## Additional file

## Pre-publication history

The pre-publication history for this paper can be accessed here:

http://www.biomedcentral.com/1741-7015/12/118/prepub

## Supplementary Material

Additional file 1: **Patients leading the direction of clinical research.** An interview with Paul Wicks. Click here for file
